# Identification of strata for a trial of a targeted multimodal physiotherapy intervention in patellofemoral pain patients

**DOI:** 10.1186/1745-6215-16-S2-P158

**Published:** 2015-11-16

**Authors:** Chris Sutton, Paola Dey, Jessie Janssen, Michael Callaghan, Jim Richards, Maria Stokes, Denis Martin, John Dixon, Erik Witvrouw, Russell Hogarth, Vasilios Baltzopoulos, Elizabeth Ritchie, Nigel Arden, Rich Masters, Remco Polman, David Turner, James Selfe

**Affiliations:** 1 University of Central Lancashire, Preston, Lancashire, UK; 2 University of Manchester, Manchester, UK; 3 University of Southampton, Southampton, Hampshire, UK; 4 Teesside University, Middlesbrough, Teesside, UK; 5 Ghent University, Ghent, Belgium; 6 Aspetar, Doha, Qatar; 7 Brunel University, London, UK; 8 Harrogate and District National Health Services, Harrogate, N Yorkshire, UK; 9 University of Oxford, Oxford, Oxfordshire, UK; 10 University of Waikato, Hamilton, New Zealand; 11 Bournemouth University, Bournemouth, Dorset, UK; 12 University of East Anglia, Norwich, Norfolk, UK

## Background

Patellofemoral Pain (PFP) is a musculoskeletal disorder causing significant pain and dysfunction around the knee, commonly leading to long term limitations. People with PFP are commonly referred for physiotherapy, although current multimodal physiotherapy approaches tend to be rather ad-hoc and are failing in the long term. Identification of patient strata (or subgroups) who may respond differentially to interventions has been recognised as an international priority. It has been proposed that there are PFP patient strata, classified based on clinical tests, who would respond to modes of interventions targeted at the individual's stratum.

## Methods

Participants with PFP underwent clinical assessment of muscle weakness, muscle length, patellar mobility and foot posture. The presence of strata was explored using two classification techniques: hierarchical cluster analysis and latent profile analysis (LPA); for LPA, the Bayesian Information Criterion was used to guide model selection.

## Results

One hundred and twenty seven (of 130) recruited participants had complete assessment data. A three strata solution appeared optimal from modelling and clinical perspectives. The three suggested strata were characterised as: stronger; weaker and tighter; weaker with pronated feet. However, although membership of the stronger stratum was consistent, there were substantial differences in the other composition of the other two strata between the two classification approaches.

## Conclusion

PFP patients can be classified into clinical strata, although further investigation is needed to identify thresholds upon which stratification should be performed. Interventions are currently being mapped onto the strata and feasibility and evaluation trials of these interventions should then be performed.

